# Superantigens Increase the Survival of Mice Bearing T Cell Lymphomas by Inducing Apoptosis of Neoplastic Cells

**DOI:** 10.1371/journal.pone.0015694

**Published:** 2010-12-22

**Authors:** Juliana Mundiñano, Paula M. Berguer, Gabriel Cabrera, Daniela Lorenzo, Irene Nepomnaschy, Isabel Piazzon

**Affiliations:** 1 ILEX-CONICET, División Medicina Experimental, Instituto de Investigaciones Hematológicas, Academia Nacional de Medicina, Buenos Aires, Argentina; 2 Consejo Nacional de Investigaciones Científicas y Técnicas, Fundación Instituto Leloir, Buenos Aires, Argentina; Health Canada, Canada

## Abstract

Superantigens bind to major histocompatibility complex class II molecules and interact with T cells expressing a particular T cell receptor Vβ inducing a strong proliferation/deletion response of the superantigen-reactive T cells. However, there have been no attempts to investigate the ability of Sags to induce apoptosis in neoplastic T cells by signaling through the Vβ region of their TCR. In the present study we show that bacterial and MMTV-encoded superantigens induce the apoptosis of AKR/J cognate lymphoma T cells both *in vitro* and *in vivo*. The Fas-Fas-L pathway was shown to be involved in the apoptosis of lymphoma T cells induced by bacterial superantigens. *In vivo* exposure to bacterial superantigens was able to improve the survival of lymphoma bearing mice. Moreover, the permanent expression of a retroviral encoded superantigen induced the complete remission of an aggressive lymphoma in a high percentage of mice. The possibility of a therapeutic use of superantigens in lymphoma/leukemia T cell malignancies is discussed.

## Introduction

Superantigens (Sags) are bacterial and viral proteins that share the ability to activate a large number of normal T cells. Sags bind to major histocompatibility complex (MHC) class II molecules as unprocessed proteins and subsequently interact with a high number of T cells expressing particular T cell receptor (TCR) Vβ chains [1]–[4]. After several rounds of proliferation, Sag reactive T cells undergo apoptosis or become anergic [2]–[4].

Bacterial Sags are a well described family of secreted protein toxins produced mainly by *Staphylococcus aureus* and *Streptococcus pyogenes* [5]. The capacity of bacterial Sags to induce the activation and deletion of T cells expressing T cell receptors (TCR) with a specific subset of TCR β-chain variable (Vβ) regions in mice has been extensively studied [2]–[3]. For instance, in mice, the staphylococcal enterotoxins (SEs) A and E engage T cell receptors bearing Vβ 3, 7, and 17, albeit with different avidities. SEB and its close sequence relatives, SECs 1-3, share reactivity with T cells bearing members of the mouse Vβ 3, 7 and 8.1-3 family [6]–[7]. We have recently described that SEI significantly stimulates mouse T cells bearing Vβ 3, 5 and 13 [8].

Mouse mammary tumor virus (MMTV) is a type B retrovirus which induces mammary adenocarcinomas in mice [9]–[10]. MMTV has two routes of infection in mice; susceptible strains acquire the virus through milk-borne infection, while other strains inherit endogenous copies of the provirus (Mtvs). For a review, see Simpson E [11]. Mtvs are present in the germline of most of the inbred mice and there are multiple proviral sequences found at different chromosomal locations in different mouse strains. Although the majority of these endogenous proviral sequences do not produce viral particles because of mutations in their regulatory or coding regions, all of them express a Sag which is encoded in their LTR region [12]–[13]. Different exogenous and endogenous proviruses cause the deletion of different classes of Vβ-bearing T cells, because they encode Sag proteins with different C-terminal aminoacid sequences [14]–[16]. We have described two variants of exogenous MMTVs, termed MMTV BALB14 and MMTV BALB2. The former encodes for a Sag which is specifically recognized by T cells bearing the Vβ14 region; MMTV BALB2 encodes for a Sag which contacts with Vβ2+ T cells [16]–[17].

Sags [18]–[20] and targeted Sags [21]–[28] have been used to enhance immunogenicity of murine and human tumor cells in different experimental models, mostly by fusing the Fab region of tumor-reactive monoclonal antibodies with mutated SEA or SEB. However, there have been no attempts to investigate the ability of Sags to induce apoptosis in neoplastic T cells by signaling through the Vβ region of their TCR.

In the present study we show that MMTV-encoded and bacterial Sags are able to induce both *in vitro* and *in vivo* the apoptosis of AKR/J spontaneous lymphoma T cells expressing cognate TCR Vβ chains. Remarkably, we show that *in vivo* exposure to Sags is able to significantly improve the survival of mice bearing cognate lymphoma T cells.

## Materials and Methods

### Mice and lymphomas

Male and female AKR/J mice bred in our animal facilities (ILEX-CONICET, División Medicina Experimental, Instituto de Investigaciones Hematológicas, Academia Nacional de Medicina) were maintained untreated until they developed spontaneous T cell lymphomas at >6 months of age. Mice were sacrificed when thymus enlargement was evident. One- to 3-mo-old male and female AKR/J mice were used as hosts of sex-matched lymphoma cells or as donors of splenocytes or macrophages. Two- to 14-mo-old AKR/J mice without thymus enlargement nor a skewed TCRVβ repertory were used to determine the level of expression of Fas, Fas-L and Bcl-2 molecules on thymocytes by fluorescence-activated cell sorting (FACS).

The mice were housed according to the policies of the ILEX-CONICET, Academia Nacional de Medicina based on *Guide for Care and Use of Laboratory Animals. Bethesda, MD: National Institutes of Health; 1985. NIH publication N.85-23.*

All experiments were approved by the ethical committee of the ILEX-CONICET (Permit number 1008).

Lymphomas were characterized by FACS in order to determine the expression of CD4, CD8, the TCRβ chain and the Vβ region, Fas, Fas-L and Bcl-2. Lymphoma cells were maintained by intraperitoneal passages in AKR/J mice. All lymphomas maintained their phenotype. Four T cell lymphomas expressing the Vβ region in >95% of the cells were chosen to investigate their reactivity to Sags: 1) T14, bearing the Vβ14 region and expressing both CD4 and CD8 molecules; 2) T8, reactive with anti-Vβ8.1,8.2 monoclonal antibody (MoAb) and expressing both CD4 and CD8 molecules; 3) T8.2, reactive with anti-Vβ8.1,8.2 MoAb and expressing the CD4 co-receptor and 4) T5, a CD4+ lymphoma expressing the Vβ5 region.

### Monoclonal antibodies

The following MoAbs conjugated to fluorescein isothiocyanate (FITC), phycoerythrin (PE) or Cy-chrome 5 (all from Pharmingen) were used for FACS analysis: anti-CD4 (clone H129.19); anti-CD8a (clone 53-6.7), anti-TCRβ (clone H57-597), anti-Vβ14 (clone 14-2), anti-Vβ17^a^ (clone KJ23), anti-Vβ13 (clone MR12-3), anti-Vβ12 (clone MR11-1), anti-Vβ11 (clone RR3-15), anti-Vβ10b (clone B21.5), anti-Vβ9 (MR10-2), anti-Vβ8.3 (clone 1B3.3), anti-Vβ8.1,8.2 (clone MR5-2), anti-Vβ7 (clone TR310), anti-Vβ6 (clone RR4-7), anti-Vβ5.1,5.2 (clone MR9-4), anti-Vβ4 (clone KT4), anti-Vβ3 (clone KJ25) or anti-Vβ2 (clone B20.6), anti-Fas (CD95 clone Jo2) and anti-Fas-L (CD178, clone MFL3). Intracellular staining of Bcl-2 was performed using FITC- or PE-conjugated anti-Bcl-2 (clone 3F11) and the Cytofix/Cytoperm kit (both from Pharmingen) according to the manufacturer's protocol.

### Flow cytometric staining

For double or triple staining, 1×10^6^ cells were incubated with the appropriate MoAbs as previously described [29]. Acquisition of 30,000 cells was performed using a FACScan flow cytometer (BD Biosciences). Results were analyzed using Cell Quest software (BD Immunocytometry Systems).

### Toxins and MMTV infection

Purified staphylococcal enterotoxin B (SEB) and E (SEE) were purchased from Toxin Technologies. Staphylococcal enterotoxin I (SEI) was a kind gift from Dr. E. Malchiodi. The toxins were diluted in PBS and kept frozen in aliquots at −20°C until use.

AKR/J mice were infected with MMTV BALB2 or MMTV BALB14 by footpad injection of 50 µl of virus-containing milk, as previously described [16]. Non-infected control mice were footpad inoculated with 50 µl of virus-free milk.

### Cultures

Different numbers of lymphoma cells were co-cultured with mytomicin-C pretreated splenocytes from MMTV-infected or non-infected AKR/J mice. Alternatively, lymphoma cells were co-cultured with intra-peritoneal macrophages obtained from thioglycolate-injected young AKR/J mice in the presence of 10 µg/ml of SEB, SEE, SEI or PBS. All cultures were performed in 96-well flat-bottom microculture plates (Corning Costar) in RPMI 1640 (Invitrogen Life Technologies) supplemented with 10% fetal bovine serum, 1% L-glutamine, 1% antibiotic-antimycotic and 50 µM 2-mercaptoethanol (Gibco, Invitrogen Life Technologies) and incubated in humidified 5% CO_2_ atmosphere at 37°C.

### Proliferation assays

The *in vitro* proliferative response of lymphoma cells to Sags was determined by the incorporation of 3H-thymidine (PerkinElmer) into DNA. Lymphoma cells were co-cultured with MMTV infected splenocytes or with macrophages exposed to bacterial Sags. At 24 hours of culture, cells were pulsed with 1 µCi of 3H-thymidine and 18 hours later were harvested on a glass fiber filter. Samples were counted in a scintillation beta-counter (Becton Dickinson).

Additionally, lymphoma cells were labelled using 5,6 carboxifluorescein diacetate succinimidyl ester (CFSE) (Molecular Probes) and cultured with Sags. After 48 hours, cells were recovered and proliferation was analyzed by CFSE dilution [30].

For *in vivo* proliferation assays, CFSE-stained lymphoma cells were intraperitoneally inoculated in AKR/J mice. One hour later mice received intraperitoneally 25 µg of SEB, SEI, SEE or PBS. Alternatively, CFSE-stained lymphoma cells were intraperitoneally inoculated in MMTV-infected and non-infected AKR/J mice. At different time intervals, cells were recovered from the intraperitoneal cavity and analyzed by FACS.

### Apoptosis assays

Lymphoma cells were cultured with 10 µg/ml of SEB, SEI, SEE o PBS. At different time intervals, cells were recovered, washed with PBS at 4°C, resuspended in 150 µl of Annexin V Binding Buffer and stained with 1 µl of Annexin V and 1 µl of 7AAD (all from Pharmingen). For DNA content analysis, propidium iodide (PI) (Sigma) was used as described previously [29].

For *in vivo* apoptosis assays, CFSE-stained lymphoma cells were intraperitoneally inoculated in AKR/J mice and one hour later mice received intraperitoneally 25 µg of SEB, SEI, SEE or PBS. Alternatively, CFSE-stained lymphoma cells were intraperitoneally inoculated in MMTV infected and non-infected AKR/J mice. Forty eight hours later cells were recovered from the intraperitoneal cavity and stained with Annexin V.

Additionally, lymphoma cells were injected in the footpad and 3 to 6 days later, 10 µg of bacterial Sags or PBS were footpad inoculated. Twenty four hours later, the draining popliteal lymph nodes (PLN) were excised and apoptosis was assessed by FACS using PI or by histology using the terminal deoxynucleotidyl transferase (TdT)-mediated dUTP nick-end labelling (TUNEL) assay. TUNEL was performed using the ApopTag In Situ Apoptosis Detection Kit (Chemicon).

### Assessment of mitochondrial membrane depolarization

To analyze changes in mitochondrial membrane potential (Δψm) by FACS, lymphoma cells cultured during 72 hours with 10 µg/ml of SEE, SEB or PBS were stained with 3,3′-diethyloxacarbocyanine iodine (DiOC_2_(3)) (Molecular Probes) at a final concentration of 10 nM according to the manufacturer's protocol. Increases in the percentage of DiOC_2_(3)^low^ cells were considered as indicative of mitochondrial depolarization. As a positive control, cells were treated in parallel samples with the protonophore uncoupling agent carbonyl cyanide 3-chlorophenylhydrazone (CCCP) (50 µM).

### Inhibition of Sag induced apoptosis

Lymphoma cells were cultured with or without 10 µg/ml of bacterial Sags. At day 2, mouse Fas-Fc protein (10 µg/ml) or human IgG (10 µg/ml) (both from Sigma) was added. Cells were collected at day 4 and apoptosis was measured using Annexin V-7AAD double staining.

For caspase inhibition assays, Z-IETD-FMK caspase-8 inhibitor or Z-LEHD-FMK caspase-9 inhibitor (Calbiochem) was added one hour prior to addition of Sags. Caspase inhibitors were used at a final concentration of 25 µM in DMSO. DMSO (0.25%) diluted in RPMI-1640 was used as vehicle control. At day 3, cells were collected and apoptosis was measured using Annexin V-7AAD double staining. The percentage of cells with mitochondrial depolarization was determined using DiOC_2_(3).

### Survival experiments

To investigate whether bacterial Sags were able to improve the survival of mice carrying cognate lymphoma T cells, 5×10^3^ T5 or T8 lymphoma cells were inoculated into the tail vein of AKR/J mice. At days 2 and 3 the mice were intraperitoneally treated with 50 µg of SEI or PBS. In another set of experiments, AKR/J mice were intravenously inoculated with 1×10^3^ T8.2 or T14 lymphoma cells. At days 2 and 3 the mice were intraperitoneally treated with 50 µg of SEB or PBS.

In order to investigate the effect of MMTV-encoded Sags, 1×10^3^ T14 lymphoma cells were intravenously inoculated in MMTV BALB2-, MMTV BALB14-infected or non-infected AKR/J mice. In another set of experiments T14-carrying mice were MMTV infected 3 days after lymphoma cell inoculation.

All survival studies were conducted in a blind and random fashion. Animals were monitored daily for general appearance and weight change. Mice showing signs of pain and suffering were killed.

### Statistical analysis

Levels of significance were determined using the two-tailed Student's t test.

Statistical analysis of TCRVβ gene product representation in normal thymocytes compared to thymic lymphoma cells was performed using the exact binomial test.

Comparison of survival curves was performed using the Log-rank test with Prism software. Survival rates were analyzed by chi-square test.

## Results

### Expression of Vβ chains in spontaneous AKR/J thymic lymphomas

Taking into account that it has been proposed that TCR engagement by endogenous Sags would be one epigenetic factor that contributes to the selection of murine pre-neoplastic T cell clones [31] we first investigated whether lymphomas bearing Vβ chains that recognize endogenous Sags were overrepresented in spontaneous AKR/J lymphomas.

**Figure 1:**
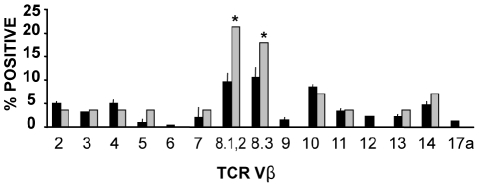
Phenotypic characterization of spontaneous thymic lymphomas. The expected versus observed frequency of spontaneous thymic lymphomas expressing specific Vβ determinants. Single cell suspensions from individual AKR/J lymphomas or from the thymus of 3 mo-old AKR/J normal mice were stained with a panel of anti-Vβ specific MoAbs and analyzed by FACS. The black bars represent the expected frequency calculated as the mean ± standard deviation of the percentage of thymocytes from 3 mo-old AKR/J normal mice (n = 5) that bind a particular anti-Vβ MoAb. The grey bars represent the observed frequency of lymphomas that bind the indicated anti-Vβ MoAb (n = 28). Asterisks mark the Vβ determinants that were represented on lymphomas at significantly greater than expected frequencies (* p<0.05).

We found that twenty eight of 38 (74%) AKR/J spontaneous lymphomas expressed TCRβ chains as detected by FACS. Six of 28 lymphomas expressing the TCRβ chain were not recognized by the panel of MoAbs used. Two thymic lymphomas were found to be Vβ10+, two Vβ14+, one Vβ3+, one Vβ4+ and one Vβ2+. Five lymphomas were Vβ8.3+. Six were recognized by the anti-Vβ8.1,8.2 MoAb. This group could include lymphomas expressing the Vβ8.1 chain which recognize an AKR/J endogenous Sag. Three lymphomas expressing other Vβ chains which recognize endogenous Sags were found (Vβ5, Vβ11 and Vβ7 respectively). Figure 1 shows the expected vs observed frequency of lymphomas expressing the indicated Vβ specificities. Expected values were based on the percentage of thymocytes in normal young AKR/J mice that express the indicated Vβ chains [31]. Neither group showed a significant overrepresentation, except the group recognized by anti-Vβ8.3 MoAb and the group recognized by anti-Vβ8.1,8.2 MoAb. It is to note that only the group of lymphomas recognized by the Vβ8.1,8.2 MoAb may include lymphomas specific for an AKR/J endogenous Sag. Thus, our results do not show a clear overrepresentation of lymphomas expressing Vβ chains recognizing endogenous Sags, and do not provide convincing support for a role of endogenous Sags in the selective expansion of pre-neoplastic clones.

### Superantigens increase the proliferation of lymphoma T cells carrying a cognate Vβ chain

**Figure 2:**
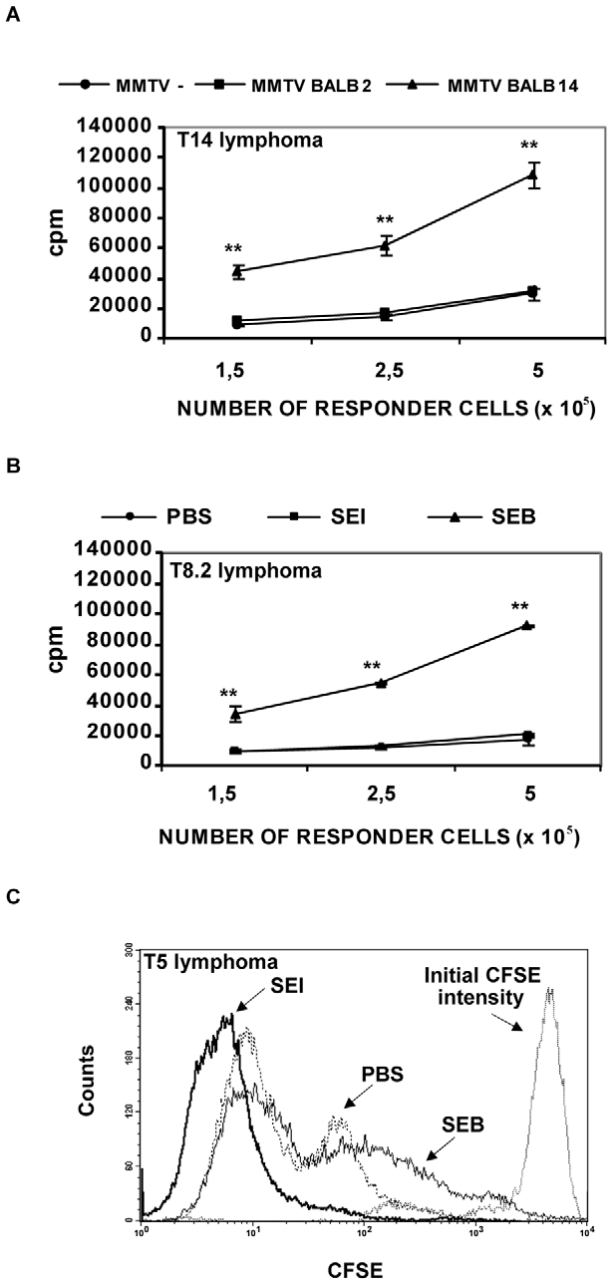
Sags induce increases in the proliferative levels of cognate lymphoma T cells. (A–B) Proliferative response of lymphoma T cells *in vitro*. (A) Different numbers of T14 lymphoma cells were co-cultured with 0.7×10^5^ mitomycin C-pretreated splenocytes from (•) non-infected AKR/J mice, (▪) MMTV BALB2-infected AKR/J mice or (▴) MMTV BALB14-infected AKR/J mice. The proliferative response was assessed by 3H-thymidine incorporation during the last 18 hr of a 1-day culture period. Data are expressed as the mean ± SD, n = 5, **p<0.01. (B) Different numbers of T8.2 lymphoma cells were co-cultured with 0.7×10^5^ mitomycin C-pretreated intraperitoneal macrophages in the presence of 10 µg/ml of (▪) SEI, (▴) SEB or (•) PBS. The proliferative response was assessed by 3H-thymidine incorporation during the last 18 hr of a 1-day culture period. Data are expressed as the mean ± SD, n = 5, **p<0.01. (C) Proliferative response of lymphoma T cells *in vivo*. CFSE-stained T5 lymphoma cells (5×10^6^) were transferred intraperitoneally into syngeneic AKR/J mice. The mice were divided into three groups (n = 3 per group) and received an intraperitoneal injection of 25 µg of SEB, SEI or PBS. Twenty four hours later cells were collected by intraperitoneal washing and CFSE fluorescence was analyzed. Overlayed histograms depict a representative example. All the experiments were performed three times with similar results.

The ability of bacterial and viral Sags to increase the *in vitro* proliferation of cognate lymphoma T cells was first investigated. T8 and T8.2 (Vβ8.1,8.2+) lymphoma cells, but not T14 (Vβ14+) and T5 (Vβ5+) neoplastic cells, strongly proliferated *in vitro* in the presence of SEB which interacts with the Vβ8 family of mouse TCRs. No alterations in the proliferative level of the Vβ8+ lymphoma cells were detected in the presence of SEI which was recently shown to interact with the Vβ5 chain in mice [8]. T5 cells increase their proliferative level in the presence of SEI. T14 cells showed a significant increase in their proliferative levels when co-cultured with splenocytes expressing the Sag encoded by MMTV BALB14 [17] whereas no alterations were detected when these cells were co-cultured with splenocytes infected with MMTV BALB2 which encodes for a Sag specific for Vβ2+ T cells [17]. Figure 2A-B depicts representative results.

In order to assess whether Sags were able to increase the *in vivo* proliferation of cognate T cell lymphomas, T5 or T8 neoplastic cells were stained with CFSE and intraperitoneally inoculated in AKR/J mice. One hour later, mice were intraperitoneally inoculated with SEI, SEB, SEE or PBS. Twenty four or 48 hours later, cells were recovered from the intraperitoneal cavity and analyzed by FACS. The decrease in the mean fluorescence intensity was significantly higher when mice were treated with Sags reactive with the Vβ expressed by the neoplastic cells (Figure 2C depicts a representative result). In some experiments, neoplastic cells recovered from mice treated with control Sags displayed a lower decrease in CFSE intensity than that observed in cells from PBS-treated mice (Figure 2C). This fact could be due to cell cycle arrest induced by toxin-dependent increases in endogenous glucocorticoids [32]–[33] or to other toxic effects [34].

These results show that Sags increase the proliferation of cognate lymphoma T cells both *in vitro* and *in vivo*.

### Superantigens induce apoptosis of cognate lymphoma T cells

The ability of Sags to induce apoptosis in cognate lymphoma T cells after 2–4 days of culture was investigated using propidium iodide or Annexin V staining. On the one hand, SEB but not SEI was able to induce a significant increase in the level of apoptosis in T8 and T8.2 cells. On the other hand, SEI but not SEB was able to increase the levels of apoptosis in T5 cells.

**Figure 3:**
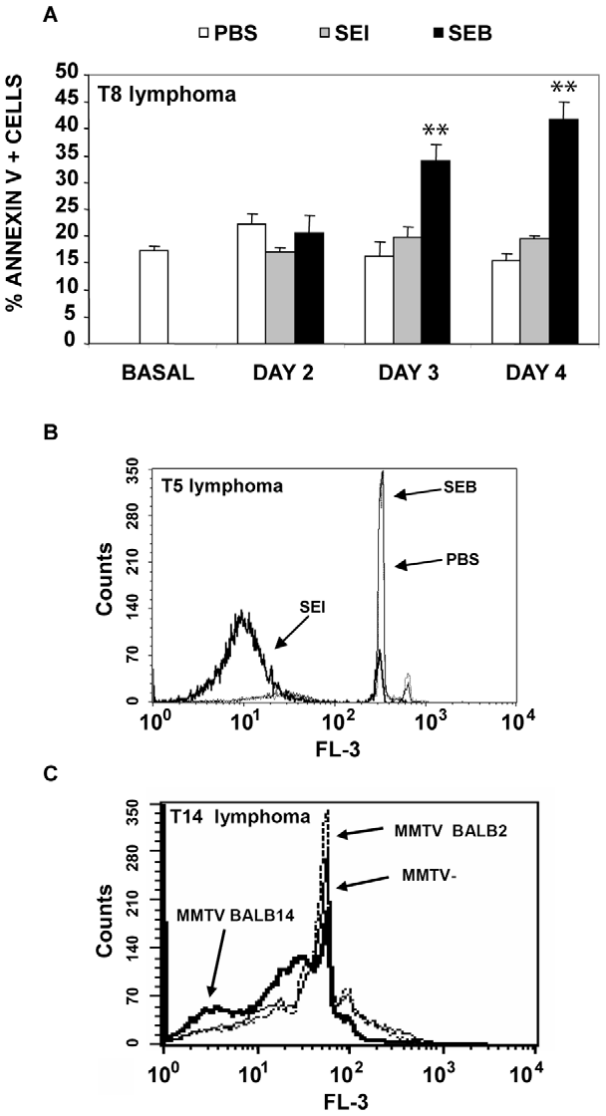
Sags increase apoptosis of cognate lymphoma T cells *in vitro*. (A and B) T8 or T5 lymphoma cells (2.5×10^5^) were co-cultured with 0.7×10^5^ intraperitoneal macrophages in the presence of 10 µg/ml of the indicated bacterial Sags or PBS. (A) Percentage of apoptosis in T8 cells at different days as assessed by FACS using Annexin V-7AAD. Data are expressed as the mean ± SD, n = 4, ** p<0.01. (B) Representative overlayed histograms of PI showing the DNA content in T5 cells cultured with 10 µg/ml of SEB, SEI or PBS during 3 days. (C) T14 lymphoma cells (2.5×10^5^) were co-cultured with 0.7×10^5^ mytomicin-C pretreated splenocytes from (MMTV-) non-infected AKR/J mice, MMTV BALB2- or MMTV BALB14-infected AKR/J mice. The Figure depicts representative overlayed histograms of PI showing the DNA content in T14 cells at day 3 of culture. All the experiments were performed five times with similar results.

Finally, splenocytes from MMTV BALB14 infected mice but not splenocytes from MMTV BALB2 infected mice were able to induce increases in the level of apoptosis in T14 cells. Figure 3A-C shows representative results.

These results show that Sags induce apoptosis in cognate lymphoma T cells *in vitro*.

**Figure 4:**
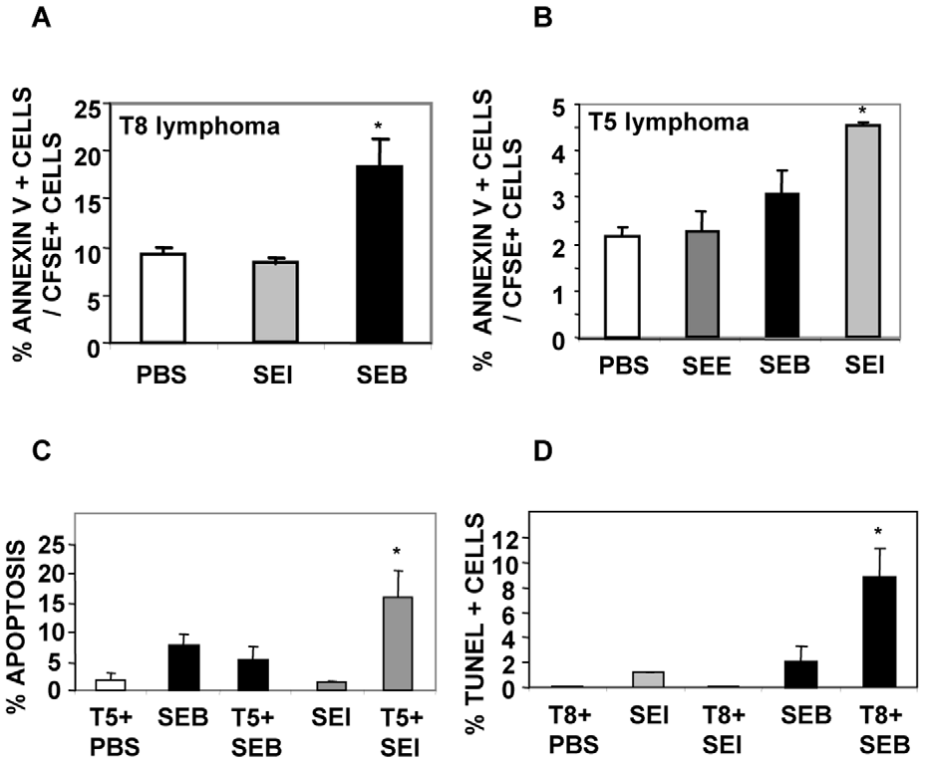
Sags increase apoptosis of cognate lymphoma T cells *in vivo*. (A–B) AKR/J mice were intraperitoneally inoculated with 5×10^6^ CFSE-labeled T8 or T5 cells. One hour later 25 µg of SEE, SEI, SEB or PBS were intraperitoneally administered. After 48 hours, cells were recovered and the percentage of Annexin V+ cells/CFSE+ cells was assessed by FACS. Data are expressed as the mean ± SD, n = 4 per group. *p<0.05. (C) AKR/J mice were inoculated in the footpad with 0.3×10^6^ T5 cells and 6 days later mice were footpad injected with 10 µg of the indicated Sag or PBS. Twenty four hours later PLN were removed and the percentage of apoptotic cells was determined by evaluating the percentage of hypodiploid nuclei by PI staining. Data are expressed as the mean ± SD, n = 4 per group. * p<0.05. (D) AKR/J mice were inoculated in the footpad with 0.3×10^6^ T8 cells and 3 days later they were treated with 10 µg of the indicated Sag or PBS. Normal AKR/J mice were also inoculated with Sags or PBS. Twenty four hours later PLN were removed and paraffin-embedded. PLN were processed for apoptosis detection using a peroxidase-conjugated *in situ* TUNEL assay. Quantitative studies were performed in a blinded fashion using light microscopy on five or more fields in each sample. Original magnification ×400. Data are expressed as the mean percentage of TUNEL-positive cells ± SD, n = 4 per field. * p<0.05. All the experiments were performed three times and gave similar results.

In order to determine whether Sags were able to induce apoptosis of lymphoma cells *in vivo*, CFSE-stained T5 or T8 cells were intraperitoneally inoculated in AKR/J mice followed 1 hour later by inoculation of SEI, SEB, SEE or PBS. Forty eight hours later cognate lymphoma T cells showed significant increases in the apoptosis levels as observed by FACS analysis (Figure 4A-B).

Additionally, T5 or T8 cells were inoculated in the footpad and 3–6 days later mice received SEI, SEB or PBS. Popliteal lymph nodes were excised 24 hours later and apoptosis was assessed by FACS and TUNEL assays. As can be observed in Figure 4C-E, a significant increase in the apoptosis of cognate lymphoma T cells was recorded.

These results show that Sags induce apoptosis in cognate lymphoma T cells not only *in vitro* but also *in vivo*.

### Apoptosis of lymphoma T cells induced by bacterial Sags involves Fas-Fas-L interaction

There are two main pathways to apoptotic cell death. One involves the interaction of a death receptor such as the tumor necrosis factor receptor-1 (TNFR1) or the Fas receptor with its ligand and the second pathway depends on the participation of mitochondria. Proapoptotic and antiapoptotic members of the Bcl-2 family regulate the mitochondrial pathway. For a review, see Arnold R. et al [35].

**Figure 5:**
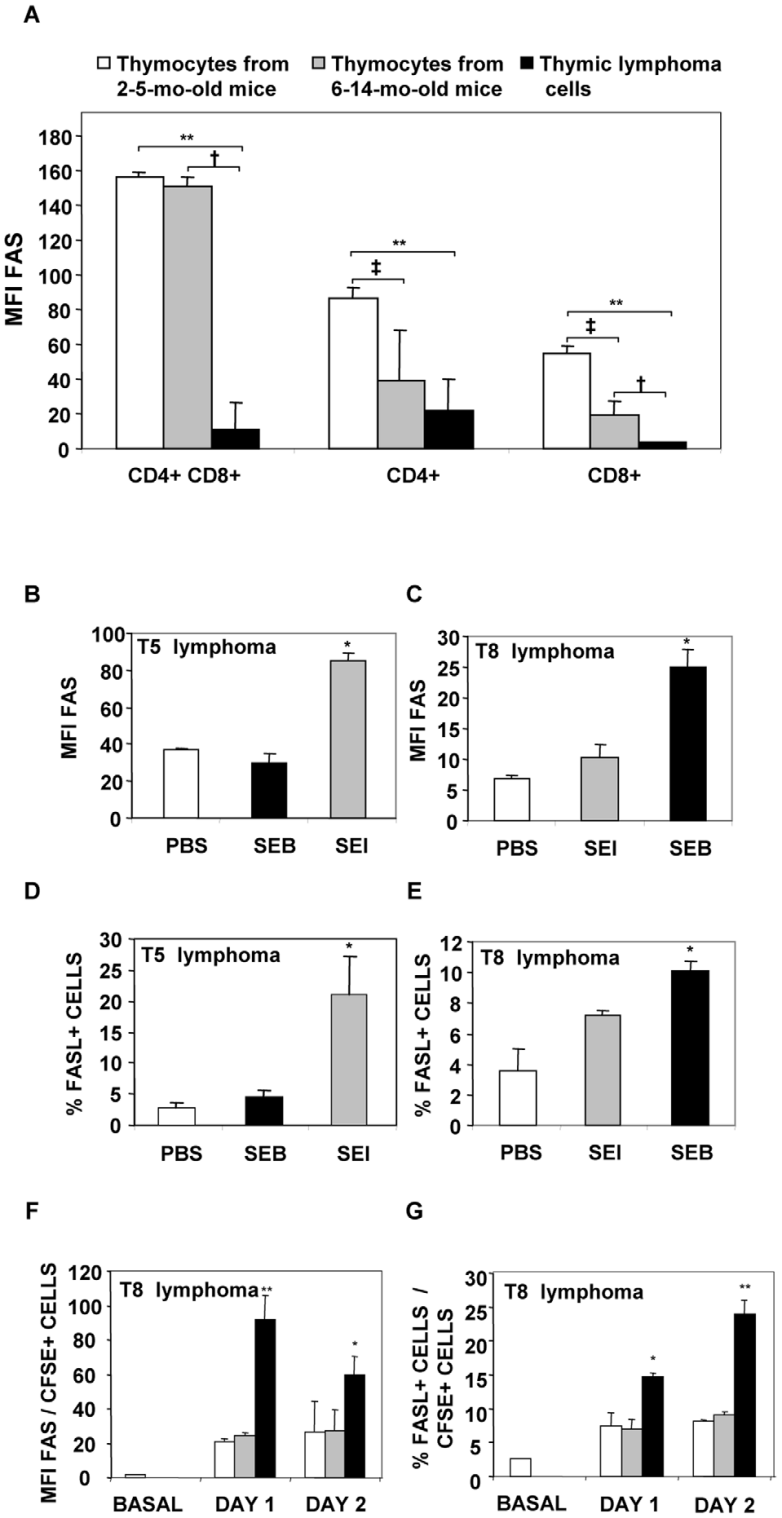
Sags induce increases in the level of expression of Fas and in the percentage of Fas-L+ cells in lymphoma T cells. Lymphoma T cells display low levels of Fas expression. (A) Single cell suspensions from thymus from 2-5-mo-old animals (n = 8), 6-14-mo-old animals (n = 11) and spontaneous AKR/J thymic lymphomas (n = 28) were stained with anti-CD4, anti-CD8 and anti-Fas MoAbs. Data show the mean fluorescence intensity (MFI) of Fas in each group. Data are expressed as the mean ± SD. **p<0.01, ^†^ p<0.05, ^‡^ p<0.05. (B, C, D, E) 2.5×10^5^ T5 or T8 lymphoma cells were co-cultured with 0.7×10^5^ intraperitoneal macrophages and treated with 10 µg/ml of SEI, SEB or PBS. At day 3, cells were harvested and stained with anti-Fas or anti-FasL MoAbs. (B, C) MFI of Fas, (D, E) percentage of FasL+ cells. Data are expressed as the mean ± SD. *p<0.05. (F, G) AKR/J mice were intraperitoneally inoculated with 5×10^6^ T8 CFSE- labeled cells and intraperitoneally injected with 25 µg of SEE (grey bar), SEB (black bar) or PBS (white bar). At days 1 and 2 cells were recovered, stained with anti-Fas or anti-FasL MoAbs and analyzed by FACS. Data are expressed as the mean ± SD, n = 4 per group. **p<0.01, *p<0.05. All the experiments were performed three times with similar results.

Whereas Bcl-2 expression was not found to be significantly increased in lymphoma cells (data not shown) Fas expression was found to be markedly decreased in the 28 lymphomas tested (Figure 5A).

The mechanisms underlying apoptosis induced by bacterial Sags in two of the lymphomas (T8 and T5) were studied. Exposure to specific bacterial Sags did not induced alterations in Bcl-2 expression or in the level of expression of TNFR1 and tumor necrosis factor-related apoptosis-inducing ligand (TRAIL) (data not shown). Contrarily, a marked increase in the expression of Fas and in the percentage of Fas-L positive neoplastic cells was recorded when cells were cultured with specific Sags (Figure 5B-E). Occasionally *in vitro* exposure of lymphoma cells to non specific bacterial Sags induced a slight but not significant increase in the expression of Fas (Figure 5E).

In order to study whether Sags were able to induce increases in Fas and Fas-L expression *in vivo*, CFSE-stained T5 and T8 lymphoma cells were intraperitoneally inoculated and 1 hour later mice received SEE, SEI, SEB or PBS. One to three days later, cognate lymphoma cells exhibited significantly higher expression levels of Fas; the percentage of Fas-L positive cells was also significantly increased. Figure 5F-G shows representative results. These results show that bacterial Sags upregulate the expression of Fas and Fas-L on lymphoma T cells expressing the appropriate TCR Vβ chain both *in vitro* and *in vivo*.

**Figure 6:**
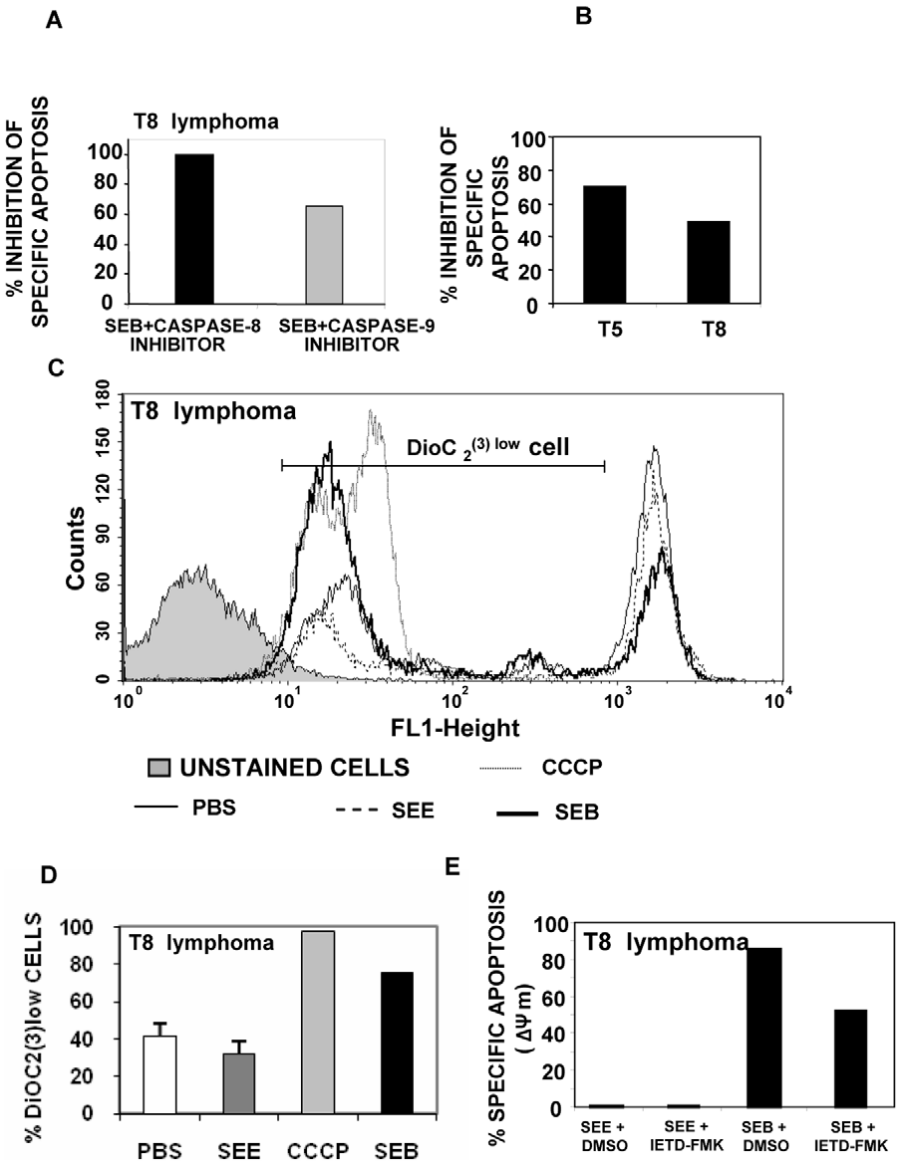
Mechanisms involved in Sag-induced apoptosis. A) Inhibition of Sag-induced apoptosis of lymphoma cells by caspase-8 and caspase-9 inhibitors. T8 cells (2.5×10^5^) co-cultured with peritoneal macrophages (0.7×10^5^) were treated during two hours with 25 µM of Z-IETD-FMK (caspase-8 inhibitor), Z-LEHD-FMK (caspase-9 inhibitor) or DMSO prior to the addition of 10 µg/ml of SEB or PBS. At day 3, cells were recovered, stained with Annexin/7-AAD and analyzed by FACS. The percentage of specific apoptosis was calculated as follows: 100× (% Sag induced apoptosis - % spontaneous apoptosis with PBS)/(100 - % spontaneous apoptosis with PBS). Inhibition of apoptosis by caspase inhibitors was calculated as reduction of specific apoptosis compared to specific apoptosis in the presence of DMSO set as 100%.(B) Inhibition of apoptosis by Fas-Fc fusion protein. T5 or T8 cells (2.5×10^5^) co-cultured with peritoneal macrophages (0.7×10^5^) were treated with SEI, SEB (10 µg/ml) or PBS. Ten µg/ml of Fas-Fc, HuIgG or PBS were added at day 2. At day 4, cells were harvested and stained with Annexin V-7AAD. Specific apoptosis was calculated as described in (A). Inhibition was calculated as reduction of apoptosis compared to apoptosis in the presence of HuIgG set as 100%. (C–D) Sags cause mitochondrial depolarization in cognate lymphoma T cells. T8 lymphoma cells (2.5×10^5^) were cultured in the presence of 10 µg/ml of SEE, SEB or PBS as described in Material and Methods. Cells were harvested 72 hours after Sag exposure and the mitochondrial membrane potential (Δψm) was measured by FACS using DiOC_2_(3) staining. Representative overlayed histograms for DiOC_2_(3) fluorescence are depicted in (C).(D) Show the percentage of DiOC_2_(3)^low^ cells. Data are expressed as the mean ± SD. *p<0.05. (E) Caspase-8 inhibitor decreases the loss of Δψm induced by SEB. T8 lymphoma cells (0.25×10^6^) were incubated with 25 µM Z-IETD-FMK or DMSO for 2 hours before exposure to 10 µg/ml of SEE, SEB or PBS. Cells were harvested 72 hours later and Δψm was measured by FACS using DiOC_2_(3) staining. Apoptotic cells were identified by their decrease in Δψm (DiOC_2_(3)^low^). Data show the percentage of specific apoptosis calculated as described in (A). All the experiments were performed at least three times with similar results.

An important inhibition of specific apoptosis could be observed when T8 cells were treated with SEB in the presence of caspase-8 inhibitor (Figure 6A). Similar results were obtained when T5 cells were cultured with SEI in the presence of caspase-8 inhibitor (data not shown). Finally, mouse Fas-Fc protein was used as a competitive inhibitor of Fas/Fas-L interactions [36]. We found that Fas-Fc effectively inhibited apoptosis (Figure 6B) confirming the involvement of the Fas-Fas-L pathway in apoptosis induced by bacterial Sags.

An involvement of mitochondria has also been demonstrated in Fas signaling, suggesting a functional role of this organelle in death receptor-mediated apoptosis of certain cell types. For a review, see Barnhart B.C. et al [37]. Changes in the membrane potential Δψm of T8 after Sag treatment was assessed using DioC_2_(3). SEB but not SEE was able to induce a significant increase in the percentage of DioC_2_(3)^low^ T8 cells (Figure 6C-D). Besides, when cells were treated with the caspase-9 inhibitor, a significant inhibition of specific cell death was recorded (Figure 6A). These results suggest that mitochondria are also involved in Sag-induced apoptosis. Finally, the caspase-8 inhibitor was able to significantly decrease the dissipation of mitochondrial Δψ (Figure 6E), raising the possibility that in our experimental conditions, Sag-induced apoptosis involves a cross-talk between the extrinsic and the mitochondrial pathways.

### Sags significantly prolong the survival of lymphoma carrying mice

**Figure 7:**
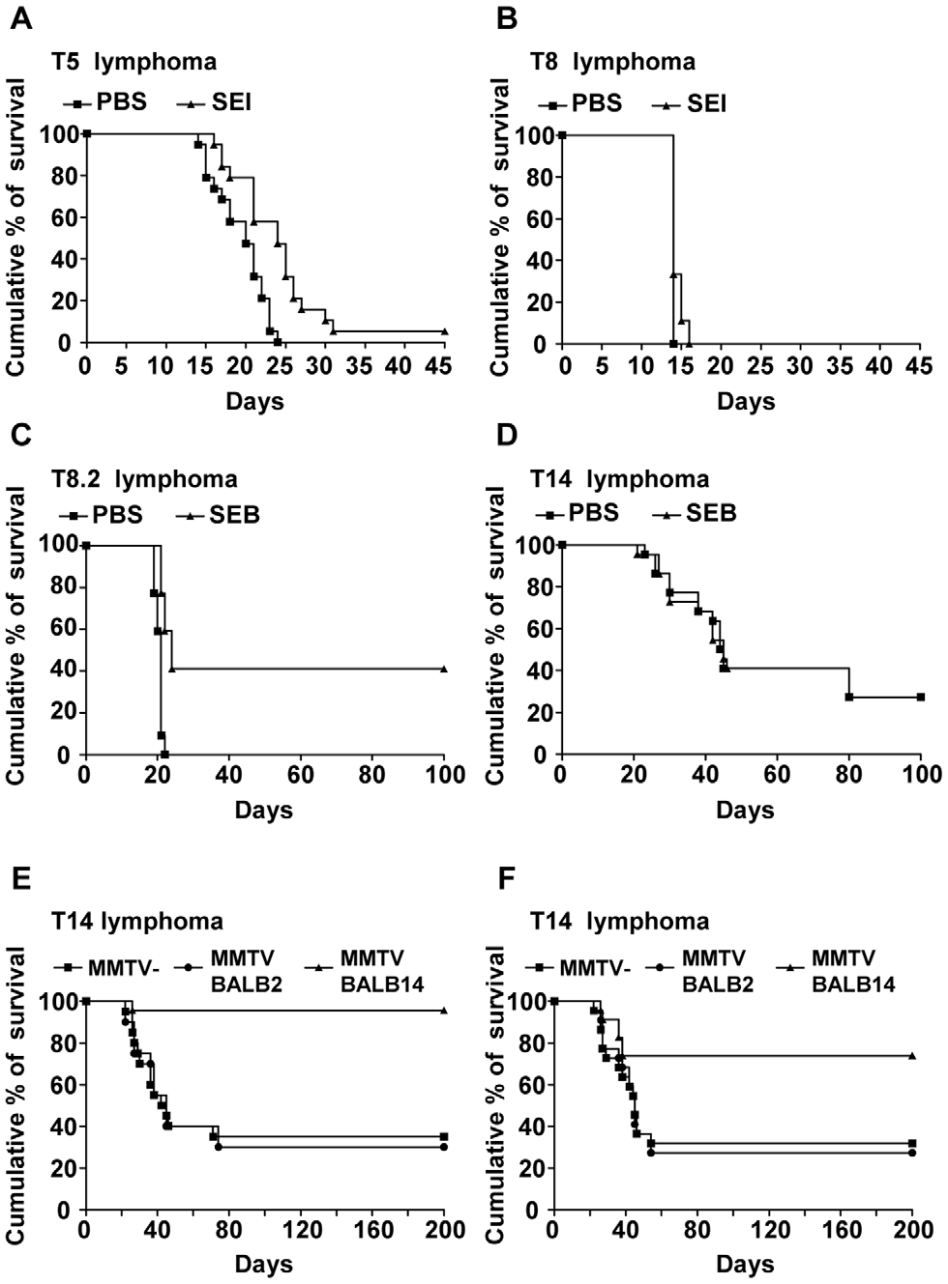
Sags increase the survival of cognate lymphoma-bearing mice. (A)T5 cells (5×10^3^) or (B) T8 cells (5×10^3^) were injected into the tail vein of AKR/J mice (n = 19 per group). At days 2 and 3, mice received an intraperitoneal injection of 50 µg of SEI or PBS. p = 0.0005, log-rank test. (C) T8.2 cells (1×10^3^) or (D) T14 cells (1×10^3^) were intravenously inoculated in AKR/J mice (n = 22 per group). At days 2 and 3 mice were intraperitoneally inoculated with 50 µg of SEB or PBS. Control mice were treated with PBS. p<0.0001, log-rank test. (E) T14 cells (1×10^3^) were injected into the tail vein of MMTV BALB14-infected (n = 22) and MMTV BALB2-infected (n = 20) and non-infected (n = 20) AKR/J mice. p<0.0001, log-rank test. (F) T14 cells (1×10^3^) were intravenously injected in AKR/J mice. Two days later, mice were infected as described in Materials and Methods with MMTV BALB14 (n = 22) or MMTV BALB2 (n = 22). Control mice received PBS (n = 22). p = 0.0123, MMTV- vs MMTV BALB14; p = 0.0075, MMTV BALB2 vs MMTV BALB14, log-rank test.

Intravenous injection of AKR/J lymphoma cells in 2 mo-old syngeneic mice caused disseminated and fatal lymphoma/leukemia. In order to evaluate whether Sags were able to increase the survival of lymphoma carrying mice T8.2, T8, T5 and T14 lymphomas were used. In a first set of experiments, AKR/J mice were intravenously inoculated with T5 or T8 cells and treated with SEI or PBS at days 2 and 3 after tumor inoculation. Figure 7A-B shows that SEI treatment was able to significantly increase the survival time of T5 lymphoma bearing mice (log-rank test, p = 0.0005). SEI did not affect the survival of mice bearing T8 cells.

In a subsequent set of experiments, AKR/J mice were intravenously inoculated with T14 or T8.2 cells. Two and 3 days later the mice were intraperitoneally treated with SEB or PBS. Figure 7C-D shows that treatment with SEB was able to significantly increase the survival of mice challenged with T8.2 lymphoma (log-rank test, p<0.0001). No significant improvement was observed in SEB-treated T14 carrying mice (log-rank test, p = 0.8). At day 22, 100% (22/22) of non-treated mice had died. After a follow-up period of 100 days, 40.9% (9/22) of SEB-treated mice survived without signs of disease (Chi-square test, p = 0.0008). Consistent with elimination of the neoplastic cells in Sag-treated mice, histopathologic and cytofluorometric examination of liver, bone marrow, lymph nodes and spleen did not reveal signs of disease (data not shown).

The effect of an MMTV-encoded Sag on the survival of cognate lymphoma T cells was also investigated. Normal or MMTV-infected AKR/J mice were intravenously inoculated with T14 cells. The 65% (13/20) of non-infected control mice died. A similar outcome was observed in MMTV BALB2-infected hosts (70%, 14/20). A significantly improved survival of MMTV BALB14 infected mice was observed, as reflected by survival of >90% (21/22) of the animals at day 200 (Chi-square test, p<0.0001), (Figure 7E). The surviving mice were killed and their lymphoid organs were studied using histopathological and cytofluometric techniques. The percentage of Vβ14+ cells in peripheral blood, lymph nodes, and spleen was found to be <0.01% in all the survivors. Two of the mice presented new thymic lymphomas, both expressing the Vβ8 family chain.

Finally, AKR/J mice intravenously inoculated with T14 lymphoma cells were infected 3 days later with either MMTV BALB2 or BALB14. As can be shown in Figure 7F, infection with MMTV BALB14 induced a significant increase in the percentage of the surviving mice: 72.7% (16/22) vs 31.8% (7/22), (Chi-square test, p = 0.0066). MMTV BALB2 infection did not alter the survival of T14 bearing mice (27.3%, 6/22) (Chi-square test, p = 0.7). All surviving mice showed less than 0.01% of Vβ14+ cells at day 200 (data not shown).

These results show that Sags are able to significantly prolong the survival of mice carrying lymphoma T cells bearing cognate Vβ chains. Moreover, the permanent expression of a retroviral-encoded Sag was able to induce the complete remission of the neoplasia in a high percentage of mice.

## Discussion

It has been extensively reported that the interaction of Sags with normal T lymphocytes expressing particular TCR Vβ chains leads initially to the stimulation and subsequently to the clonal deletion by apoptosis of reactive T cells [1]–[4]. However, there is scarce information concerning the effects of Sags on cognate neoplastic T cells.

Herein we show that bacterial and MMTV-encoded Sags are able to induce both *in vitro* and *in vivo* the apoptosis of AKR/J lymphoma T cells bearing cognate Vβ chains. Importantly, Sags significantly improve the survival of mice carrying cognate T cell lymphomas.

A role for Sags in the progression of murine T cell lymphomas has been hypothesized. It has been proposed that TCR engagement by endogenous Sags would be one epigenetic factor that contributes to the evolution and selection of murine neoplastic T cell clones [31]. To test this hypothesis we investigated whether lymphomas bearing Vβ chains that recognize endogenous Sags were overrepresented in spontaneous AKR/J lymphomas. We found that both the frequency of Vβ8.1,8.2+ lymphomas (which could include lymphomas expressing the forbidden Vβ8.1 chain) and that of Vβ8.3+ lymphomas (carrying a Vβ segment that does not recognize any endogenous Sag in AKR/J mice) were significantly increased. Tumors expressing other Vβ chains were not found to be significantly overrepresented. Thus, our results failed to provide convincing evidence for a role of endogenous Sags in the selective expansion of pre-neoplastic clones.

One important issue addressed in this study is whether AKR/J neoplastic T cells are able to undergo apoptosis upon exposure to Sags. Although Sags increased the proliferative levels of lymphoma T cells expressing a cognate Vβ chain both *in vitro* and *in vivo*, proliferation was followed/accompanied by death by apoptosis of cognate neoplastic T cells. Sags did not induce increases in the apoptosis levels of non cognate lymphoma cells. These results show that Sags are able to signal through the Vβ chain and to induce apoptosis in murine lymphoma T cells both *in vitro* and *in vivo*. Interestingly, our results show that a lymphoma that escaped thymic negative selection driven by an endogenous viral Sag (Mtv-9), and thus expresses a forbidden Vβ chain (Vβ5), is still susceptible to the induction of apoptosis triggered by a bacterial Sag reactive with its TCR. In this sense, it has been proposed that different Sags may form distinct ternary MHC-Sag-TCR T cell signaling complexes which could lead to distinct physiological outcomes [38].

Different mechanisms of apoptosis induced by bacterial Sags in normal T cells have been reported. Several groups have reported Fas-Fas-L independent apoptosis pathways after stimulation with bacterial Sags. These pathways include members of the Bcl-2-family [39]–[40] and/or reactive oxygen species [41]. Other studies, however, have shown that Fas/Fas-L interaction is involved in bacterial Sag mediated apoptosis [36], [42]–[43]. Finally, it has been proposed that the pathway involved would depend on the doses of bacterial Sags or on the experimental model used. After determining that the expression of Bcl-2 in the AKR/J spontaneous lymphomas was not altered while Fas expression was markedly decreased in all thymomas tested, the mechanisms underlying apoptosis induced by bacterial Sags in two lymphomas were studied. Whereas Sag exposure did not induce alterations in the expression of Bcl-2, TRAIL or TNFR1, interaction of Sags with the cognate Vβ chain of lymphoma T cells revert the low expression of Fas characteristic of these cells both *in vitro* and *in vivo*. Inhibition of caspase-8 markedly decreased apoptosis induced by Sags. Mouse Fas-Fc- a competitive inhibitor of Fas-Fas-L interactions effectively inhibited apoptosis in the lymphomas studied confirming the involvement of the Fas-Fas-L pathway in apoptosis induced by bacterial Sags. It has been reported that in certain cell types mitochondria is involved in Fas signaling. For a review, see Barnhart B.C. et al [37]. Herein we show that Sags induced loss of transmembrane potential in this organelle. Besides, a significant inhibition of apoptosis was recorded in the presence of a caspase-9 inhibitor. Finally, inhibition of caspase-8 significantly decreased the dissipation of Δψm, raising the possibility that Sag-induced apoptosis involves a cross talk between the extrinsic and the mitochondrial pathways.

Finally, the *in vivo* effect of retroviral and bacterial Sags on the survival of lymphoma carrying mice was investigated. SEI was able to significantly increase the survival time of mice carrying T5 neoplastic cells. Treatment with SEB was able to induce long-term survival of hosts injected with T8.2 neoplastic cells. Whereas all control mice died in three weeks, 40% of SEB treated mice were still free of disease at day 100. The fact that toxins did not affect the survival of mice bearing non-cognate lymphoma T cells strongly suggests that the effect of these toxins is mainly due to their superantigenic activity.

The effect of a retroviral-encoded Sag in the survival of lymphoma bearing mice was assessed. Mice intravenously inoculated with T14 cells showed a remarkable enhancement of survival when the hosts were infected with MMTV BALB14 before or after tumor inoculation. No improvement in survival was observed in MMTV BALB2 infected hosts or in mice treated with SEB. These results clearly show that Sags are able to significantly improve the survival of mice bearing cognate-T cell lymphomas.

The fate of the interaction between Sags and human lymphoma/leukemia T cells is a matter of debate. It has been reported that leukemic cells from patients with leukemia of T cell origin have the ability to respond to TCR-dependent bacterial Sags, as assessed by their proliferative response in vitro [44]–[45]. Based on these data, it has been proposed that bacterial infection in such patients might contribute to the expansion of leukemic cells. Noteworthy, apoptosis of leukemic cells was not assessed. It has also been hypothesized that bacterial Sags could be involved in malignant transformation and/or in the expansion and evolution of cutaneous T cell lymphomas (CTCL), this hypothesis was based in the restricted use of particular Vβ by CTCL cells [46]–[48]. Contrarily, Vonderheid EC et al [49] hypothesized that chronic stimulation of skin-homing normal T cells by staphylococcal Sags would act to deplete Vβ-responsive normal T cells prior to neoplastic transformation and as a consequence the Vβ usage by the neoplastic T cells may be skewed and they would be more likely to express Vβ segments that are relatively unaffected by staphylococcal Sags. It would be of great interest to determine whether different human T cell malignancies expressing TCRs are susceptible to the induction of apoptosis by Sags. Our unpublished results show that cells from the human Jurkat T cell line derived from an acute T cell leukemia are highly susceptible to Sag-induced apoptosis (I.N. and I.P., manuscript in preparation).

Results reported herein clearly show that cells from AKR/J T cell lymphomas are susceptible to be deleted as a consequence of Sag signaling via TCR. Importantly, Sags were able to increase the survival of mice bearing very aggressive lymphomas. If Sags were able to induce apoptosis in human neoplastic T cells expressing functional TCRs, they could be envisaged as therapeutic agents. The use of Sags as therapeutic agents in T cell malignancies would have the advantage of deleting restricted T cell clones without causing the death of other normal cells. If toxic Sags are to be used in humans, their toxic effects might be separated from their superantigenic activity. In this sense, it has been shown that carboxymethylation of SEB blocks its enterotoxic but not its mitogenic properties [50]. Furthermore, many retroviral-encoded Sags are not associated with toxic effects and human T cells are able to recognize them, opening the possibility of their use in gene therapy. Finally, treatments based on the inoculation of dendritic cells transfected with mRNA coding for MMTV Sags would avoid both toxicity and the risks associated with genetic therapies.
